# Dysphagia optimized knowledge‐based planning for head and neck cancer

**DOI:** 10.1002/acm2.70519

**Published:** 2026-02-24

**Authors:** Tu Thi, Kirk Luca, Justin Roper, Ben Hopkins, Eduard Schreibmann, Austin Smith, James Bates, Bill Stokes, Amit Jethanandani, Soumon Rudra, Xiaofeng Yang, Shadab Momin

**Affiliations:** ^1^ Department of Radiation Oncology Emory University Atlanta Georgia USA; ^2^ Medical Dosimetry Program Southern Illinois University Carbondale Illinois USA; ^3^ Department of Physics and Astronomy Vanderbilt University Nashville Tennessee USA

**Keywords:** constrictor muscles, dysphagia, head and neck cancer, knowledge‐based planning, treatment planning, volumetric modulation radiation therapy

## Abstract

**Purpose:**

Swallowing dysfunction after radiotherapy (RT) is often linked to pharyngeal mucosal damage. This study aimed to develop a dysphagia‐optimized knowledge‐based planning (DO‐KBP) model by incorporating individual pharyngeal constrictors (DO‐KBP) into an existing model, which was based on a conventional approach of including pharynx as a single structure during treatment planning (P‐KBP).

**Materials and methods:**

The P‐KBP model was trained on 175 head and neck cases with the pharynx contoured as a single organ. The DO‐KBP model included 36 additional oropharynx cases (∼20% increase) with individual pharyngeal constrictors delineated. Both models were evaluated on 25 test patients. Treatment plans generated by each model were normalized to planning‐target‐volume (PTV) coverage (D95%), and dosimetric parameters were compared using two‐tailed paired *t*‐tests. A blind physician review assessed clinical preference.

**Results:**

The DO‐KBP model was able to significantly reduce the mean dose to the inferior constrictor (36.52 ± 9.87 Gy to 19.52 ± 6.23 Gy) and superior/middle constrictors (51.89 ± 6.31 Gy to 47.46 ± 6.12 Gy) (*p* < 0.05) with the addition of 36 high‐quality treatment plans. Though statistically significant increases in mean dose were observed for the spinal cord PRV (D0.03cc), cochlea, mandible, and left brachial plexus with the DO‐KBP model, these differences were small in magnitude and remained within clinical goals. However, these differences were small in magnitude and remained within clinical goals. Plan homogeneity was equivalent (HI = 0.09). The DO‐KBP plans were preferred in blinded review.

**Conclusion:**

Targeted addition of a small number of cases with individually contoured constrictors to an existing model significantly improved sparing of swallowing structures, without compromising overall plan quality or increasing organs‐at‐risk (OAR) doses beyond clinical thresholds, highlighting that modest, focused data augmentation can yield clinically meaningful gains.

## 1 INTRODUCTION

Locally advanced head and neck cancers (HNCs) are frequently managed with curative‐intent chemoradiotherapy, where radiation therapy is a cornerstone—especially for inoperable patients. However, a major treatment‐related morbidity is radiotherapy‐induced dysphagia, which markedly impairs quality of life with approximately 60%–75% of patients developing swallowing dysfunction.^1^ Dysphagia following radiotherapy is primarily attributed to damage to the pharyngeal constrictor muscles (PCMs).^2^, ^3^ In a prior study, Peponi et al. demonstrated that sparing the swallowing structures during intensity‐modulated radiotherapy (IMRT) correlates with a significant reduction in severe long‐term dysphagia, further reinforcing the dose‐dependent impact on these critical anatomical regions.^4^

Multiple previous studies have shown that reducing radiation dose to the PCMs is associated with a lower risk of significant dysphagia.^5^, ^6^, ^7^ A recent landmark phase III trial demonstrated that dysphagia‐optimized intensity‐modulated radiotherapy (DO‐IMRT) can significantly improve swallowing function compared with standard IMRT, highlighting the clinical relevance of dose reduction to dysphagia‐associated structures.^8^ DO‐IMRT plans are optimized with the emphasis on individual pharyngeal constrictors in comparison to the conventional approach of treatment planning with pharynx as a single structure. The Quantitative Analyses of Normal Tissue Effects (QUANTEC) guidelines recommend mean dose to the entire pharynx organ to be less than 50 Gy.^9^ More modern analyses have suggested stronger correlations between dysphagia and RT dose to PCM.^8^, ^10^, ^11^ Although VMAT can precisely shape dose around the target, efforts to spare organs‐at‐risk (OARs) can introduce planning tradeoffs that may (1) shift dose to other OARs or (2) compromise tumoricidal target coverage. Incorporating each constrictor muscle as an OAR may further complicate the treatment planning process. In the case of adaptive radiotherapy to correct for treatment variations, the re‐planning may be required for up to three times for a large number of HNC patients, which further adds to the treatment planning time and complexity.^12^

Knowledge‐based planning (KBP) techniques, such as RapidPlan, offer an advanced solution by automating treatment planning through predictive modeling.^13^, ^14^ Briefly, RapidPlan KBP predicts achievable DVHs for various structures included in the model, which are then converted into optimization objectives. The algorithm consists of two main components: model configuration and DVH estimation. During model configuration, the data extraction phase gathers geometric features of each structure to prepare the dataset for training. The model training phase then develops a DVH estimation model for each structure. Following this, the DVH estimation component generates estimated DVHs and optimization objectives. While KBP shows substantial potential for automation and standardization, developing a high‐quality model remains a labor‐intensive process. It requires careful selection of representative training cases with consistent contouring and high‐quality dose distributions, particularly when additional OARs such as individual pharyngeal constrictors are included, along with expertise in model development and treatment planning. These logistical and resource demands can be especially challenging in busy clinical environments with limited personnel or competing priorities. Therefore, approaches that improve performance with initial new data are especially valuable.

Previously, KBP models investigating RT‐related dysphagia in oropharynx cancer patients incorporated the pharyngeal constrictors as a single composite structure.^15^ Including the composite structure in the model yields useful DVH predictions for the entire structure; however, it does not provide spatial information regarding the distribution across the individual pharyngeal musculature. We hypothesized that including individual pharyngeal constrictors in the training phase of KBP would provide more desirable dose distribution due to the additional spatial and geometrical features of individual constrictors encoded within KBP model. This study aimed to develop an enhanced KBP model (DO‐KBP) by incorporating individual pharyngeal constrictors and other dysphagia‐related structures, with the goal of improving plan quality without the need to build a new model from scratch. We demonstrate the feasibility of clinical implementation of DO‐KBP model by comparing its performance against an HN model with the pharynx as a single OAR (P‐KBP). We pursued this via a small, targeted augmentation by adding only 36 oropharynx cases to an existing, clinically validated model.

## 2 METHODS

### 2.1 Patient selection

Twenty‐five patients treated with chemotherapy in combination with RT for oropharyngeal cancer requiring bilateral neck radiation at our institution between 2020 and 2021 were selected for this study. All patients received a prescription dose of 70 Gy in 35 fractions to the gross disease, with a simultaneous integrated dose to the regional nodal lymph node chains, consistent with recently published consensus guidelines.^16^ The gross tumor volume (GTV), encompassing the gross disease, was expanded by 5 mm to generate the high‐risk clinical target volume (CTV_7000) and then anatomically modified according to barriers to spread. CTV_7000 was then expanded by 3 mm to create the planning‐target‐volume (PTV_7000). In addition to PTV_7000, the intermediate and low dose PTVs consisted of elective/low risk nodes. All PTV volumes were cropped 3 mm from the skin surface. The institutional review board (IRB) approval was obtained for this study.

### 2.2 Model generation and validation

A previously published KBP model for HNC patients receiving bilateral neck RT was used as a benchmark for plan quality and dosimetric evaluation.^15^ This KBP model (P‐KBP) was developed using 175 clinically approved treatment plans, all of which met institutional planning objectives and represented a diverse range of HNC cases. The P‐KBP model was trained using the entire pharynx structure as a single organ to receive a mean dose less than 50 Gy. To incorporate pharyngeal constrictors, 36 additional high‐quality, clinically approved oropharyngeal treatment plans were added to the P‐KBP model, resulting in the development of the DO‐KBP model. The clinical plans all used the original P‐KBP model as a starting point for these 36 training cases, and further manual optimization was required to account for pharyngeal constrictors. This updated model explicitly specifies the superior/middle and inferior pharyngeal constrictors as separate structures, reducing their mean doses to 50 and 20 Gy, respectively. The anatomical boundary between the superior/middle and inferior pharyngeal constrictors is defined at the caudal extent of the hyoid bone.^8^ All other aspects of the model and workflow were unchanged.

**FIGURE 1:**
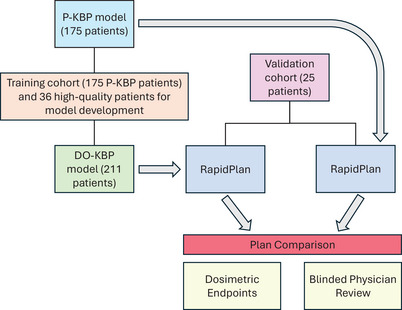
Schematic of the training and validation workflow for P‐KBP and DO‐KBP models.

The DO‐KBP model was validated by comparing its dose distributions to those from clinical data, dose–volume histogram (DVH) estimation bands, and optimized plans were compared to clinically treated plans. This validation process was conducted using 25 oropharyngeal patients plans that were not included in model training. Selected plans were replanned using the DO‐KBP model to refine dose predictions for specific OARs identified as outliers. Both the P‐KBP and DO‐KBP models shared identical optimization constraints, except for the pharyngeal delineation. The optimization templates for each model are detailed in Appendix Tables S1, S2, and S3. Figure 1 depicts the schematic of the workflow for training and evaluation for both P‐KBP and DO‐KBP models.

### 2.3 Treatment planning

Treatment planning was performed using the Varian Eclipse treatment planning system (Version 16.1). VMAT optimization was conducted with the Photon Optimizer, and dose calculations were performed using the Anisotropic Analytical Algorithm with a 2.5 mm dose calculation grid. Each patient had two treatment plans generated (50 total plans): one using the P‐KBP model and the other using the DO‐KBP model. Importantly, none of the study plans were included in the training datasets for either KBP model. RapidPlan version 16.1.0 was used to train and apply both KBP models.

All treatment plans were optimized for a Varian TrueBeam linear accelerator with three full arcs, using collimator angles of 350°, 10°, and 90°. Optimization was conducted with convergence mode and restarting at level MR3 for the intermediate dose calculation. Within the optimizer, the appropriate KBP model was selected, target dose levels were defined, and all relevant OARs were matched to the model. Any OARs that completely overlapped with one or more PTVs were excluded from the plan optimization and analysis. Automatic optimization mode and automatic intermediate dose calculation were enabled, and no modifications were made to the optimization objectives generated by the KBP model. Following optimization and final dose calculation, all plans were normalized such that the prescription dose covered 95% of the highest prescription volume.

### 2.4 Physician review

All RT plans were blinded and reviewed by a single physician. For each case, the physician had access to the dose distribution and DVHs and was asked to select the preferred plan. The physician was informed that all plans were generated using a KBP model, but was not made aware of which model was used or the purpose of the study. The physician evaluated all aspects of treatment plan, including the doses to all OARs and target coverage.

### 2.5 Data analysis

The dose‐volume parameters used in this study are shown in Table 1, which are based on current clinical practice. Total monitor units (MU) and plan homogeneity (((D_2_‐D_98_)/D_p_)) were recorded for each plan. The D_2_ and D_98_ indicate the minimum dose received by 2% and 98% of the target volume, respectively, while D_P_ represents the prescribed dose. A two‐tailed paired *t*‐test was used to compare the dosimetric constraints of the P‐KBP and DO‐KBP models. A Wilcoxon signed‐rank test was performed to evaluate differences in total MUs between the two planning groups, with statistical significance set at *p* < 0.05.

**TABLE 1:** Summary of dose volume parameters for evaluation of both KBP models.

Structure	Dose volume parameter	Dose limit per protocol (Gy)
Spinal Cord PRV	D0.03cc (Gy)	≤50
Spinal Cord	D0.03cc (Gy)	≤45
Optic Nerve/Chiasm	D0.03cc (Gy)	≤52
Parotid L	mean (Gy)	≤26
Parotid R	mean (Gy)	≤26
Larynx	mean (Gy)	≤40
Gland Submandibular L	mean (Gy)	≤39
Gland Submandibular R	mean (Gy)	≤39
Esophagus	D0.03cc (Gy)	<60
	mean (Gy)	<30
Cavity_Oral—CTV	D0.03cc (Gy)	<60
	mean (Gy)	<30
Cochlea L	mean (Gy)	<35
Cochlea R	mean (Gy)	<35
Mandible	D0.03cc (Gy)	<73
Brachial Plexus L	D0.03cc (Gy)	≤66
Brachial Plexus R	D0.03cc (Gy)	≤66
Pharynx Inferior—CTV	mean (Gy)	<20
Pharynx Sup/middle—CTV	mean (Gy)	<50

## 3 RESULTS

**FIGURE 2:**
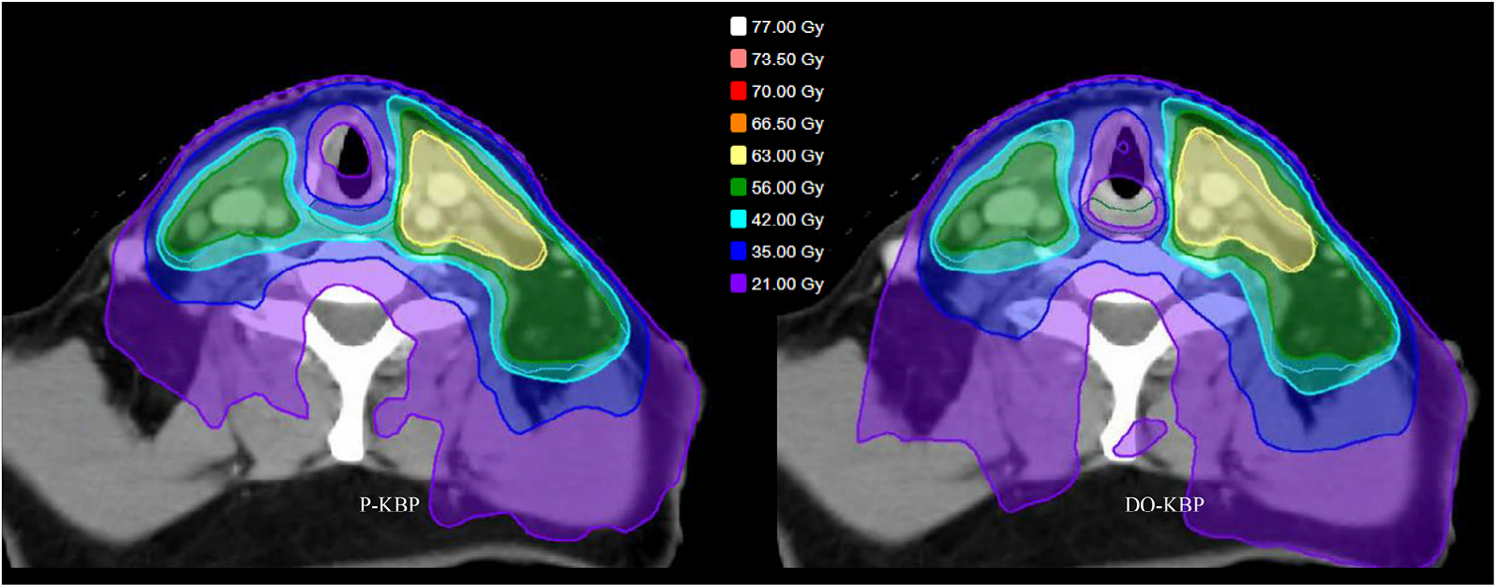
Dose distribution comparison between P‐KBP and DO‐KBP optimized plan for a sample case from validation cohort. The inferior constrictor is outlined in green.

A comprehensive qualitative and quantitative comparison was conducted between treatment plans generated by the P‐KBP and DO‐KBP models. Figure 2 presents the dose distribution for a representative validation case. The DO‐KBP plan demonstrated a notable reduction in dose to the inferior constrictor, whereas the P‐KBP plan exhibited a 35–40 Gy dose bridge extending over the inferior constrictor region.

**FIGURE 3:**
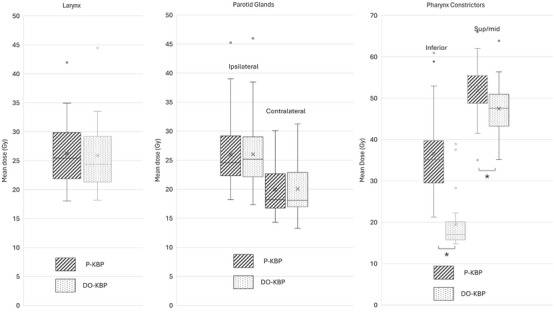
Box plot of clinically relevant dose volume metrics for larynx (left), parotid glands (middle), and pharyngeal constrictors (right) over 25 treatment plans generated by P‐KBP (line) and DO‐KBP (dotted). Significance level at p < 0.05 is indicated by asterick.

Figure 3 shows that the DO‐KBP model significantly reduced the mean dose for both the inferior and superior/middle pharyngeal constrictors. There were no significant differences in mean dose values between P‐KBP and DO‐KBP for larynx, ipsilateral parotid gland, or contralateral parotid gland.

Table 2 summarizes the dosimetric parameter values averaged over 25 validation cases, along with the statistical analysis, including paired *t*‐test and 95% confidence intervals of the differences in mean between both models. The DO‐KBP model significantly reduced D_mean_ for inferior (19.52 ± 1.25 Gy vs. 36.52 ± 1.97 Gy) and superior/middle (47.46 ± 1.22 Gy vs. 51.89 ± 1.26 Gy) constrictors compared to P‐KBP model. Across the 19 clinically relevant dosimetric parameters evaluated, the two models produced highly similar results, with the exception of the pharyngeal constrictors. The DO‐KBP model resulted in a lower D0.03_cc_ for esophagus. Statistically significant increases in mean dose were observed for the cochlea, mandible, and left brachial plexus with the DO‐KBP model. However, these differences were small in magnitude and remained within clinical goals. Additionally, the D0.03cc for the spinal cord PRV increased slightly but remained within acceptable limits.

**TABLE 2:** Comparison of dosimetric parameters mean ± standard error over 25 validation cases and their *p*‐value from two tailed paired *t*‐test between P‐KBP and DO‐KBP models.

Structure	Dose volume parameter	P‐KBP			DO‐KBP			*p*‐value	Difference of the mean CI
Spinal Cord PRV	D0.03cc (Gy)	22.62	±	0.49	24.25	±	0.54	**0.01**	[−3.20, −0.05]
Spinal Cord	D0.03cc (Gy)	16.11	±	0.44	16.68	±	0.28	0.13	[−1.34, 0.19]
Optic Chiasm	D0.03cc (Gy)	2.17	±	0.13	2.22	±	0.14	0.30	[−0.14, 0.04]
Parotid Ipsilateral	mean (Gy)	25.98	±	1.18	26.03	±	1.20	0.74	[−0.37, 0.27]
Parotid Contralateral	mean (Gy)	19.92	±	0.83	20.07	±	0.89	0.42	[−0.50, 0.21]
Larynx	mean (Gy)	26.24	±	1.18	25.91	±	1.18	0.26	[−0.22, 0.88]
Submandibular L	mean (Gy)	30.26	±	1.43	30.96	±	1.47	0.11	[−4.93, 3.53]
Submandibular R	mean (Gy)	34.39	±	1.43	34.22	±	1.35	0.57	[−3.89, 4.23]
Esophagus	D0.03cc (Gy)	37.76	±	1.78	36.56	±	1.92	0.03	[0.13, 2.28]
	mean (Gy)	14.07	±	0.75	14.35	±	0.86	0.29	[−0.81, 0.25]
Cavity_Oral—CTV	D0.03cc (Gy)	69.38	±	1.97	69.51	±	2.00	0.49	[−0.49, 0.24]
	mean (Gy)	31.53	±	1.72	31.65	±	1.70	0.52	[−0.51, 0.26]
Cochlea L	mean (Gy)	5.25	±	0.44	5.46	±	0.45	0.00	[−0.34, −0.09]
Cochlea R	mean (Gy)	4.85	±	0.47	4.95	±	0.49	0.01	[−0.18, −0.03]
Mandible	D0.03cc (Gy)	70.54	±	1.43	70.86	±	1.49	0.03	[−0.60, −0.04]
Brachial Plexus L	D0.03cc (Gy)	62.37	±	1.65	62.75	±	1.70	0.00	[−0.61, −0.16]
Brachial Plexus R	D0.03cc (Gy)	60.09	±	2.53	60.20	±	2.50	0.41	[−0.038, 0.15]
Pharynx Inf—CTV	mean (Gy)	36.52	±	1.97	19.52	±	1.25	0.00	[14.43, 19.56]
Pharynx Sup/mid—CTV	mean (Gy)	51.89	±	1.26	47.46	±	1.22	0.00	[3.62, 5.24]

Plan homogeneity was similar between the two models, with both achieving an average homogeneity index of 0.09. Additionally, the DO‐KBP plans demonstrated a statistically significant reduction in MU. The average MU for the DO‐KBP plans was 752, compared to 756 for the P‐KBP plans. During the blinded plan review, the physician selected the DO‐KBP plans for every case reviewed (25/25).

**FIGURE 4:**
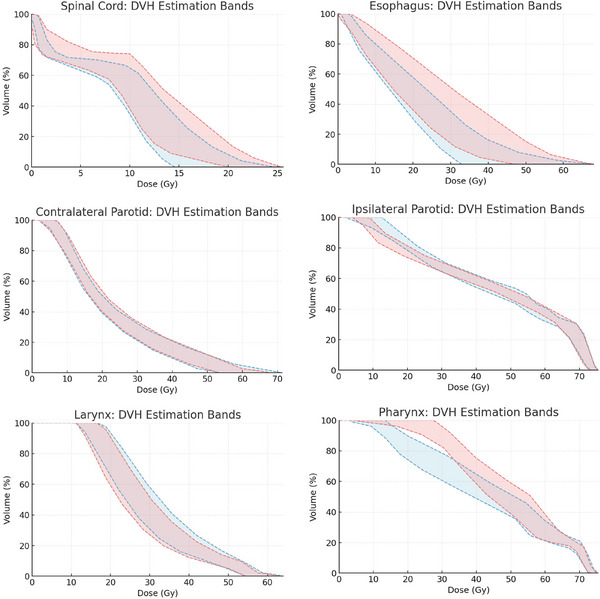
The average upper and lower DVH estimation bands for validation cases for P‐KBP model (red) and DO‐KBP model (blue).

**FIGURE 5:**
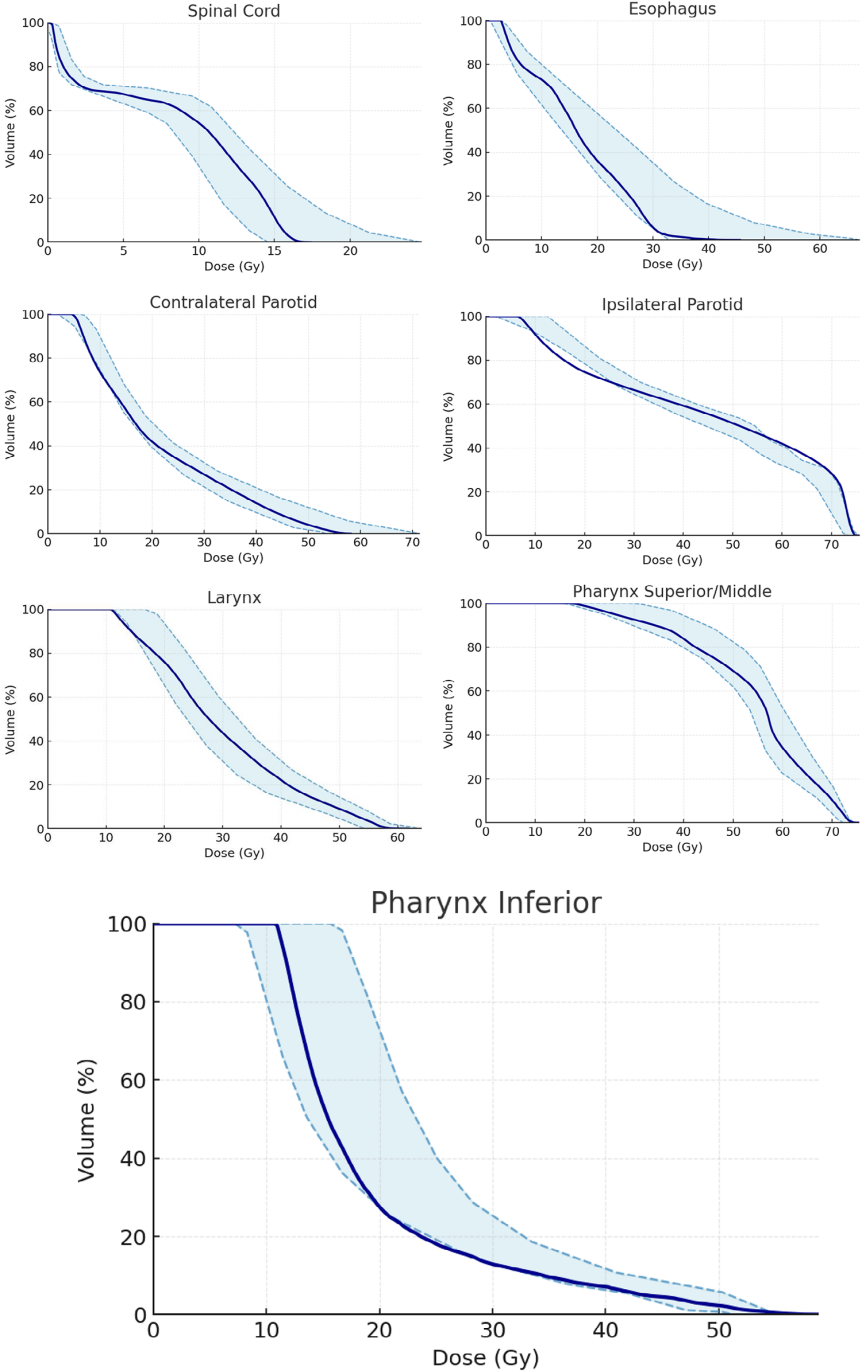
The average upper and lower DVH estimation bands for validation cases for DO‐KBP model.

To demonstrate the overall change in prediction performance in validation cases between P‐KBP and DO‐KBP models, the comparison of average upper and lower estimation bands is shown in Figure 4 for larynx, parotid glands, esophagus, spinal cord and pharynx. The DO‐KBP resulted in lower overall DVH estimations than P‐KBP model for esophagus and pharynx, whereas comparable DVH estimations were observed for spinal‐cord, parotid glands, and larynx. Figure 4 also demonstrates that the prediction accuracy for other important OARs, such as spinal cord, esophagus, larynx, and parotid glands, has been maintained after incorporating additional cases and constraints for the pharyngeal constrictors. Figure 5 shows the average of predicted upper and lower DVH bands and achieved DVHs for the test cohort for DO‐KBP model. Overall, achieved DVHs fall well within the prediction bands for DO‐KBP model, which confirms the accuracy of the predictions.

## 4 DISCUSSION

Previous research has established a correlation between pharyngeal constrictor dose and late dysphagia, which can complicate quality of life for HNC patients.^17^, ^18^, ^19^, ^20^ While patient‐reported outcomes have helped guide dose‐volume parameters to the constrictor apparatus, identifying an optimal threshold to potentially limit late dysphagia is still a work in progress.^21^ While the treatment planning for HNC commonly relies largely on dose volume recommendations made by QUANTEC,^9^ recent efforts for HNC emphasize sparing dysphagia aspiration structures surrounding the PTV. In this study, we developed a DO‐KBP model and demonstrated its potential for clinical implementation for patients treated with definitive RT for oropharynx cancer.

QUANTEC recommends limiting the mean dose to the pharyngeal structure to less than 50 Gy for HNC. This is thought to translate into a reduction of dysphagia risk by 20%. Recent works recommended dose volume constraints for individual constrictor muscles for various endpoints.^5^, ^22^, ^23^, ^24^, ^25^, ^26^ For instance, maintaining mean inferior pharyngeal constrictor dose to ≤41 Gy and V_40Gy_ ≤ 41% may help minimize gastrostomy tube dependence.^25^ In our analysis of the 50 treatment plans for 25 patients, we found that the dysphagia‐optimized KBP (DO‐KBP) model notably reduced the mean dose to the PCMs. On average, the DO‐KBP plans delivered a 10–15% lower dose to these muscles compared to the conventional KBP (P‐KBP) plans (*p* < 0.05). For instance, the DO‐KBP model was able to reduce dose to the inferior pharyngeal constrictor by an average of 17.00 Gy compared to plans generated by P‐KBP (19.52 Gy vs. 36.52 Gy), which is clinically significant. The DO‐KBP model was able to lower D_mean_ to superior/middle pharyngeal constrictors by 4.4 Gy (47.46 Gy vs. 51.89 Gy). Mean doses ≥ 50 Gy to the superior/middle PCMs are associated with late dysphagia.^23^ The improvement in pharyngeal constrictors sparing was consistent across the entire cohort, indicating that incorporating individual superior/middle and inferior constrictors as OARs effectively spares these critical swallowing structures without compromising target coverage. However, the tradeoffs of improved pharyngeal constrictor sparing resulted in a statistically significant increase in five dose‐volume parameters, including the right parotid gland, though none of these observed tradeoffs were deemed to be clinically significant. There was a modest increase in the D_max_ by about 3% for the spinal cord compared to the P‐KBP plans. It is important to note that while all the OARs were added to the DO‐KBP model, enhanced pharyngeal constrictor sparing led to a slight increased dose to other structures, including spinal cord D_0.03cc_, mandible D_0.03cc_, and left brachial plexus D_0.03cc_, which remained within clinically acceptable limits. This suggests that although the maximum dose is a bit higher, it remains confined to the target region and does not extend into the surrounding sensitive tissues.

Notably, the increased plan quality in the DO‐KBP model was achieved by incorporating only 36 additional cases into the existing P‐KBP model, demonstrating that new objectives can be effectively integrated without requiring complete model redevelopment. This streamlined approach underscores the adaptability of KBP, allowing institutions to evolve and tailor their models as clinical priorities shift such as placing greater emphasis on toxicity reduction or functional preservation—without the burden of collecting extensive new datasets. The ability to achieve meaningful clinical improvements with a relatively small addition of training data illustrates the efficiency and flexibility of KBP refinement. It is important to note that while it is possible to introduce new objectives for constrictors into the P‐KBP model, it may not reflect the possible tradeoffs in DVHs amongst the different structures that DO‐KBP would for the structures involved. Figure 4 shows these overall trade‐offs amongst different clinical structures between P‐KBP and DO‐KBP model. Another disadvantage of adding just objectives into the model rather than additional cases would be possibility of infeasible constraints. This approach presents a practical path forward for centers with limited resources, enabling them to improve model performance and adapt planning strategies as clinical guidelines shift. This finding supports the broader use of iterative KBP model updates as a viable strategy for continuous quality improvement in radiotherapy planning.

A limitation of the present study is that pharyngeal constrictors were contoured by a single observer. Contouring the pharyngeal constrictors presents challenges due to their complex anatomy, subtle borders, and significant inter‐observer variability, which may lead to inconsistent delineation and potential miscalculation of radiation doses between the pharyngeal constrictors. While it was beyond the scope of this study to measure inter‐observer variability, this limitation can be addressed by contouring of pharyngeal constrictors on CT imaging taking into account the information derived from magnetic resonance (MR) images.^27^ Though the sample size was relatively small for validation cases, it was within a range shown to be adequate for a KBP model for HNC radiation therapy.^14^ Although this study did not include a robust evaluation of segmentation quality due to its nature, various strategies—such as percent overlap of observer volumes and robust atlas‐based methods—can be clinically employed to ensure high‐quality segmentation of the pharyngeal constrictors.

All treatment plans were generated within a single institution, limiting variations in contouring and dosimetric protocols seen across multiple institutions. To enhance the model's robustness and account for differences among planners and dosimetry protocols, validation using multi‐institutional data is necessary. Furthermore, the current training model included only oropharynx cases receiving bilateral neck RT. The current model evaluated oropharynx cancer cases with the inferior and superior/middle pharyngeal constrictors, achieving mean dose constraints of 20 and 50 Gy, respectively. The performance of the current model on the single‐sided HNC cases will be evaluated in future work. While KBP models have been built for HNC, it has not been widely adopted for the purpose of pharyngeal constrictor sparing. Overall, these results demonstrated the ability of the proposed DO‐KBP model to achieve significant pharyngeal constrictor sparing compared to P‐KBP model.

## 5 CONCLUSION

This study evaluated the dosimetric performance of a PCM‐focused model for HNC radiotherapy using advanced KBP techniques. The DO‐KBP model significantly reduces doses to pharyngeal constrictors, structures critical for swallowing, with negligible dosimetric tradeoffs to other healthy tissues. This approach may improve plan quality and reduce planning time and should be prospectively studied to further optimize outcomes and validate its clinical benefits. Importantly, high‐quality models can be achieved by incorporating a relatively small number of targeted training cases and applying strategic optimization objectives, avoiding the need to construct an entirely new model from the ground up.

## AUTHOR CONTRIBUTIONS

**Tu Thi**: Investigation; Writing—Original Draft. **Kirk Luca**: Investigation; Conceptualization; Methodology; Project Administration; Writing—Original Draft; Writing—Review & Editing. **Justin Roper**: Writing—Review & Editing. **Ben Hopkins**: Writing—Review & Editing. **Eduard Schreibmann**: Writing—Review & Editing. **Austin Smith**: Writing—Review & Editing. **James Bates**: Writing—Review & Editing. **Bill Stokes**: Writing—Review & Editing. **Amit Jethanandani**: Writing—Review & Editing. **Soumon Rudra**: Writing—Review & Editing. **Xiaofeng Yang**: Writing—Review & Editing. **Shadab Momin**: Investigation; Conceptualization; Methodology; Data Curation; Formal Analysis Writing—Original Draft; Writing—Review & Editing.

## CONFLICT OF INTEREST STATEMENT

Dr. James Bates: Advisory Board: Castle Biosciences.

## ETHIC STATEMENT

For this retrospective study, all methods were performed in accordance with relevant guidelines and regulations. Patient confidentiality was maintained throughout the study.

## Supporting information



Supporting information

Supporting information

Supporting information
