# Supplementary material for: Dysphagia optimized knowledge‐based planning for head and neck cancer
Source: J Appl Clin Med Phys. 2026 Feb 24;27(3):e70519. doi: 10.1002/acm2.70519 (PMC12931426; doi:10.1002/acm2.70519)
Supplement: Supplementary file 1 — Supporting information [file ACM2-27-e70519-s002.docx]

**Table S1:** Summary of dose objectives for target structures for the P-KBP and DO-KBP models

| **Structure ID** | **Volume [%]** | **Dose [%]** | **Priority** |
| --- | --- | --- | --- |
| PTV High |  |  |  |
| Upper | 0 | 105 | 200 |
| Upper | 3 | 104 | 125 |
| Upper | 0 | 105 | 200 |
| Lower | 100 | 103 | 200 |
| Lower | 100 | 103 | 200 |
| PTV Intermediate |  |  |  |
| Lower | 100 | 103 | 200 |
| Lower | 100 | 103 | 200 |
| PTV_Low |  |  |  |
| Lower | 100 | 103 | 200 |
| Lower | 100 | 103 | 200 |
| z_PTV Intermediate |  |  |  |
| Upper | 0 | 105 | 200 |
| Upper | 3 | 104 | 125 |
| Upper | 0 | 105 | 200 |
| Lower | 100 | 103 | 200 |
| Lower | 100 | 103 | 200 |
| z_PTV Low |  |  |  |
| Upper | 0 | 105 | 200 |
| Upper | 3 | 104 | 125 |
| Upper | 0 | 105 | 200 |
| Lower | 100 | 103 | 200 |
| Lower | 100 | 103 | 200 |
