# Supplementary material for: Dysphagia optimized knowledge‐based planning for head and neck cancer
Source: J Appl Clin Med Phys. 2026 Feb 24;27(3):e70519. doi: 10.1002/acm2.70519 (PMC12931426; doi:10.1002/acm2.70519)
Supplement: Supplementary file 2 — Supporting information [file ACM2-27-e70519-s003.docx]

**Table S2:** Summary of dose objectives for organs at risk

| **Structure ID** | **Volume [%]** | **Dose [%]** | **Priority** | **gEUD a** |
| --- | --- | --- | --- | --- |
| Bone Mandible |  |  |  |  |
| Upper | 0 | Generated | 125 |  |
| Mean |  | Generated | 85 |  |
| Line | Generated | Generated | 100 |  |
| Brachial Plexus Left |  |  |  |  |
| Mean |  | Generated | 80 |  |
| Brachial Plexus Right |  |  |  |  |
| Mean |  | Generated | 80 |  |
| Brain |  |  |  |  |
| Mean |  | Generated | 100 |  |
| Line | Generated | Generated | 100 |  |
| Brainstem | 0 | Generated | 150 |  |
| Mean |  | Generated | 125 |  |
| Line | Generated | Generated | 120 |  |
| Brainstem PRV03 |  |  |  |  |
| Upper | 0 | Generated | 140 |  |
| Mean |  | Generated | 120 |  |
| Line | Generated | Generated | 100 |  |
| Cavity Oral |  |  |  |  |
| Upper | Generated | 20 | 100 |  |
| Upper | Generated | 30 | 110 |  |
| Upper | Generated | 50 | 100 |  |
| Lower | Generated | 70 | 80 |  |
| Lower | Generated | 103 | 200 |  |
| Mean |  | Generated | 120 |  |
| Line | Generated | Generated | 130 |  |
| Cochlea Left |  |  |  |  |
| Mean |  | Generated | 90 |  |
| Line | Generated | Generated | 60 |  |
| Cochlea Right |  |  |  |  |
| Mean |  | Generated | 90 |  |
| Line | Generated | Generated | 60 |  |
| Esophagus |  |  |  |  |
| Upper | Generated | 25 | 90 |  |
| Upper | Generated | 30 | 90 |  |
| Upper | Generated | 50 | 90 |  |
| Upper | Generated | 70 | 90 |  |
| Mean |  | Generated | 100 |  |
| Line | Generated | Generated | 100 |  |
| Eye Left |  |  |  |  |
| Upper | 0 | Generated | 100 |  |
| Mean |  | Generated | 100 |  |
| Line | Generated | Generated | 70 |  |
| Eye Right |  |  |  |  |
| Upper | 0 | Generated | 100 |  |
| Mean |  | Generated | 100 |  |
| Line | Generated | Generated | 70 |  |
| Gland Submandibular Right |  |  |  |  |
| Mean |  | Generated | 110 |  |
| Line | Generated | Generated | 110 |  |
| Gland Submandibular Left |  |  |  |  |
| Mean |  | Generated | 110 |  |
| Line | Generated | Generated | 110 |  |
| Larynx |  |  |  |  |
| Upper | Generated | 25 | 100 |  |
| Upper | Generated | 30 | 100 |  |
| Upper | Generated | 50 | 100 |  |
| Upper | Generated | 75 | 100 |  |
| Upper | Generated | 85 | 80 |  |
| Mean |  | Generated | 110 |  |
| Line | Generated | Generated | 110 |  |
| Lens Left |  |  |  |  |
| Upper | 0 | Generated | 100 |  |
| Mean |  | Generated | 100 |  |
| Line | Generated | Generated | 70 |  |
| Lens Right |  |  |  |  |
| Upper | 0 | Generated | 100 |  |
| Mean |  | Generated | 100 |  |
| Line | Generated | Generated | 70 |  |
| Lips |  |  |  |  |
| Mean |  | Generated | 70 |  |
| Line | Generated | Generated | 70 |  |
| Optic Chiasm |  |  |  |  |
| Upper | 0 | Generated | 100 |  |
| Line | Generated | Generated | 80 |  |
| Optic Nerve Left |  |  |  |  |
| Upper | 0 | Generated | 100 |  |
| Line | Generated | Generated | 80 |  |
| Optic Nerve Right |  |  |  |  |
| Upper | 0 | Generated | 100 |  |
| Line | Generated | Generated | 80 |  |
| Parotid Left |  |  |  |  |
| Mean |  | Generated | 125 |  |
| Line | Genreated | Generated | 120 |  |
| Parotid Right |  |  |  |  |
| Mean |  | Generated | 125 |  |
| Line | Genreated | Generated | 120 |  |
| *Pharynx |  |  |  |  |
| Upper | 80 | Generated | 90 |  |
|  |  | Generated | 90 |  |
|  | Generated | Generated | 80 |  |
| **Pharynx |  |  |  |  |
| Upper gEUD |  | Generated | 100 | 1 |
| Line | Generated | Generated | 100 |  |
| **Pharynx Inferior |  |  |  |  |
| Upper gEUD |  | Generated | 150 | 1 |
| Line | Generated | Generated | 150 |  |
| **Pharynx Superior/Middle |  |  |  |  |
| Upper gEUD |  | Generated | 100 | 1 |
| Line | Generated | Generated | 100 |  |
| Spinal Cord |  |  |  |  |
| Upper | 0 | Generated | 160 |  |
| Line | Generated | Generated | 120 |  |
| Spinal Cord PRV05 |  |  |  |  |
| Upper | 0 | Generated | 150 |  |
| Line | Generated | Generated | 110 |  |
| Thyroid |  |  |  |  |
| Mean |  | Generated | 70 |  |
| Line | Generated | Generated | 70 |  |
| zPosterior Avoid |  |  |  |  |
| Mean |  | Generated | 120 |  |
| Line | Generated | Generated | 100 |  |

*Dose constraint only in the P-KBP model

**Dose constraint only in the DO-KBP model
