# Supplementary material for: Dysphagia optimized knowledge‐based planning for head and neck cancer
Source: J Appl Clin Med Phys. 2026 Feb 24;27(3):e70519. doi: 10.1002/acm2.70519 (PMC12931426; doi:10.1002/acm2.70519)
Supplement: Supplementary file 3 — Supporting information [file ACM2-27-e70519-s001.docx]

**Table S3:** Summary of normal tissue objective settings

| Priority | 250 |  |  |
| --- | --- | --- | --- |
| Distance from Target Border | 0.05 cm |  |  |
| Start Dose | 101% |  |  |
| End Dose | 25% |  |  |
| Fall-off | 0.15 mm^-1^ |  |  |
